# A Six-month Follow-up Study of Maternal Anxiety and Depressive Symptoms among Japanese

**DOI:** 10.2188/jea.18.84

**Published:** 2008-04-10

**Authors:** Yuki Sato, Tadaaki Kato, Naoko Kakee

**Affiliations:** 1 Department of Health Policy, National Research Institute for Child Health and Development.

**Keywords:** Maternal Welfare, Anxiety, Depression, Asian Continental Ancestry Group

## Abstract

**Background:**

Maternal psychological distress has been widely studied, but epidemiologic data based on follow-up studies of maternal psychological distress remain insufficient in Japan. The objective of this study was to estimate the prevalence of anxiety and depressive symptoms among child-rearing women in Japan at two time-points after childbirth.

**Methods:**

A self-administered questionnaire was delivered on two occasions to 2,657 women who had given birth in 2004: first when their infants were 3-4 months old and then again when their infants were 9-10 months old. The questionnaire included the Hospital Anxiety and Depression Scale (HADS; Japanese version) to estimate the level of maternal psychological distress.

**Results:**

The total percentage of women with anxiety symptoms as assessed by a HADS score of 8+ was 26.2 % at 3-4 months of age, and 26.1 % at 9-10 months. Among the women without anxiety symptoms at 3-4 months, 11.6 % showed anxiety symptoms at 9-10 months. The total percentage of depressive symptoms was 19.0 % at 3-4 months, and 24.0 % at 9-10 months. Among the women without depressive symptoms at 3-4 months, 14.0 % showed depressive symptoms at 9-10 months.

**Conclusion:**

Anxiety symptoms in mothers appeared to persist from 3-4 months to 9-10 months after childbirth, while depressive symptoms tended to be more common at 9-10 months after childbirth. Nevertheless, the prevalence of anxiety symptoms was higher than that of depressive symptoms.

## INTRODUCTION

Maternal psychological distress has been widely reported because it carries the risk of progression in the future to more severe symptoms and of having a negative effect on mother-infant interactions, as well as children’s health care and behavioral development.^1^^-^^4^ In Japan, the National Project entitled “Healthy Parents and Children 21” aims at significantly reducing, by the year 2010, the percentage of women who develop postpartum depression and/or anxiety.^5^^,^^6^ Although many researchers have examined these issues, basic data from follow-up studies are apparently insufficient in Japan. Additional epidemiologic data regarding maternal psychological distress are necessary for conducting research and to develop effective social approaches. The objective of this study was to examine the prevalence of maternal anxiety and depressive symptoms at two-times points after childbirth in Japan: the first when the infants are age 3-4 months old, and the second when they are 9-10 months old.

## METHODS

This survey was conducted as part of a longitudinal study focused on women’s satisfaction with childbirth and parenting; the study protocol and characteristics of the participants are described in detail elsewhere.^7^ In brief, women who had given birth between January and December of 2004 in any of the 25 collaborating birth centers were recruited before they were discharged from these centers. The collaborating birth centers comprised 4 advanced medical centers (comprehensive prenatal medical center and university hospitals), 3 public community hospitals, 5 private clinics, and 13 maternity homes (i.e., with midwife services). Women who had stillbirths or babies with severe congenital malformations, had been transferred to other hospitals, had serious medical problems after childbirth, or were unable to understand Japanese were excluded. In total, 2,657 women gave written informed consent for participation in this study. During this period, 7,500 births occurred, in total, in the collaborating births centers.

The following self-administered questionnaire survey was conducted twice: first when the infants were 3-4 months old (first survey) and then again when they were 9-10 months old (second survey). The questionnaire contained questions pertaining to the condition of the infant and the status of maternal psychological distress. These survey time-points were adapted to coincide with infant health check-ups which are planned by many local governments.

The level of maternal psychological distress was estimated using the Japanese translation of the original version of the Hospital Anxiety and Depression Scale (HADS).^8^^,^^9^ The HADS is a rapid and relatively simple screening method, allowing both the anxiety and depressive status to be easily assessed simultaneously. The Japanese version of the HADS has been validated in two investigations.^10^^,^^11^ Furthermore, the HADS has also been reported to be a reliable method for screening the psychological status of non-hospitalized populations.^11^^-^^13^ The HADS consists of 14 items, each with four-responses, and separately measures the levels of anxiety and depression. The scores for each subscale range from 0 (no symptoms) to 21 (maximum distress), with higher scores indicating more severe distress. The HADS cutoff points for anxiety and depressive symptoms are as follows; 7 or lower: normal; 8 to 10: possible level; 11 or higher: definite level.

The response rate in the first survey was 63.5 % (n = 1,688), and that in the second survey was 69.1 % (n = 1,836). Those responding to both the surveys accounted for 53.0 % of the study participants (n = 1,408). To determine the change in the prevalence of psychological distress in the same subjects between the two times surveys, only data obtained from participants who responding to both of the surveys were analyzed.

Of the 1,408 participants who responded to both the surveys, 60 who had provided incomplete responses to the questionnaire were excluded, and the remaining 1,348 participants were finally included in the analysis. McNemar’s paired test was used for comparison of proportions. All statistical tests were two-sided. A *P*-value of less than 0.05 was considered as statistically significant.

All statistical analyses were undertaken using SPSS^®^ 14.0 J for Windows.

The protocol for this study was approved by the Institutional Review Board of the National Center for Child Health and Development.

## RESULTS

The characteristics of the 1,348 women included in the analysis were: median age, 32 years (range, 17 to 45 years), 65 % were 30 to 39 years of age; 99 % had a partner, 76 % had been graduated from a university or college, 41 % had an annual household income of 4.0-8.0 million yen, the delivery was the first in 49 %, and 7 % of the infants had a birth weight of < 2500 g.

Table 1 shows the percentage of women in each of the two surveys with anxiety symptoms. The total percentage of women with HADS-anxiety scores of 8 or more (possible and definite level) was 26.2 % (n = 353) at 3-4 months of age (first survey) and 26.1 % (n = 352) at 9-10 months (second survey). The percentage of women with HADS-anxiety scores indicative of possible level was 16.5 % in the first survey and 15.5 % in the second survey, and the percentage of women with HADS-anxiety scores indicative of definite level was 9.6 % in the first survey and 10.6 % in the second survey. There were no significant differences in the status of anxiety symptoms in the women between the two surveys (*P* = 0.62). Analysis of the data revealed that 9.2 % of women with normal anxiety scores in the first survey showed scored indicative of possible level in the second survey, and 2.4 % with normal score in the first survey showed scores indicative of definite level in the second survey. That is, among the women with normal score in the first survey, 11.6 % (n = 116) showed scores indicative of possible or definite anxiety in the second survey. In addition, 18.4 % of women with scores indicative of possible level in the first survey scores indicative of definite level in the second survey.

**Table 1.** Maternal anxiety status at 3-4 months and 9-10 months of age.

Anxiety symptoms	at 3-4 months of age										

	Normal (HADS ≤ 7)			Possible level (HADS = 8-10)			Definite level (HADS ≥ 11)			Total	
			
	n	%	(column %)	n	%	(column %)	n	%	(column %)	n	%
at 9-10 months of age											
Normal (HADS ≤ 7)	879	65.2	(88.3)	99	7.3	(44.4)	18	1.3	(13.8)	996	73.9
Possible level (HADS = 8-10)	92	6.8	(9.2)	83	6.2	(37.2)	34	2.5	(26.2)	209	15.5
Definite level (HADS ≥ 11)	24	1.8	(2.4)	41	3.0	(18.4)	78	5.8	(60.0)	143	10.6

Total	995	73.8	(100.0)	223	16.5	(100.0)	130	9.6	(100.0)	1348	100.0

*P*-value in McNemar’s paired test for the difference in the total percentage between at 3-4 months and 9-10 months of age; *P*= 0.62

HADS: Hospital Anxiety and Depression Scale

Table 2 shows the percentage of women with depressive symptoms in each of the two surveys. The total percentage of women with HADS-depression scores of 8 or more (possible and definite level) was 19.0 % at 3-4 months (first survey) and 24.0 % at 9-10 months (second survey). The percentage of women with HADS-depression scores indicative of possible case was 14.9 % in the first survey and 18.2 % in the second survey, and the percentages of women with HADS-depression scores indicative of definite case was 4.1 % in the first survey and 5.9 % in the second survey. The status of depressive symptoms in these women shifted between the first and second surveys (*P* < 0.001). Analysis of the survey data revealed that 12.7 % of the women with normal scores in the first survey showed scores indicative of possible level in the second survey, and 1.3 % of the women with normal score in the first survey showed scores indicative of definite level in the second survey. Totally, among the women without depressive symptoms in the first survey, 14.0 % (n = 153) showed depressive symptoms in the second survey. In addition, 18.9 % of the women with score indicative of possible level in the first survey showed scores indicative of definite level in the second survey.

**Table 2.** Maternal depressive status at 3-4 months and 9-10 months of age.

Depressive symptoms	at 3-4 months of age										

	Normal (HADS ≤ 7)			Possible level (HADS = 8-10)			Definite level (HADS ≥ 11)			Total	
			
	n	%	(column %)	n	%	(column %)	n	%	(column %)	n	%
at 9-10 months of age											
Normal (HADS ≤ 7)	939	69.7	(86.0)	80	5.9	(39.8)	5	0.4	(9.1)	1024	76.0
Possible level (HADS = 8-10)	139	10.3	(12.7)	83	6.2	(41.3)	23	1.7	(41.8)	245	18.2
Definite level (HADS ≥ 11)	14	1.0	(1.3)	38	2.8	(18.9)	27	2.0	(49.1)	79	5.9

Total	1092	81	(100.0)	201	14.9	(100.0)	55	4.1	(100.0)	1348	100.0

*P*-value in McNemar’s paired test for the difference in the total percentage between at 3-4 months and 9-10 months of age; *P* < 0.001

HADS: Hospital Anxiety and Depression Scale

Women who had both anxiety and depressive symptoms, i.e., both HADS scores for both anxiety and depression were at least 8 but no more than 10 (possible level), accounted for 4.1 % (n = 55) of all the women in the first survey and 5.3 % (n = 72) of all the women in the second survey. Similarly, using a cutoff score of 11 or more (definite level), women with coexistent anxiety and depressive symptoms accounted for 1.9 % (n = 26) of all the women in the first survey and 2.9 % (n = 39) of all the women in the second survey.

## DISCUSSION

In this study, 26 % of the women reported anxiety symptoms at both survey time-points, that is, when their infants were 3-4 months and 9-10 months old. On the other hand, a higher percentage of women reported depressive symptoms in the second survey than in the first survey (24.0 % vs.19.0 %). Overall, the percentage of women with anxiety symptoms was higher than that of those with depressive symptoms. Other studies using the same assessment scale have reported a higher prevalence of depressive than of anxiety symptoms in the general population.^11^^,^^12^ Child-rearing women are thus more likely to have symptoms of anxiety rather than depressive symptoms until their infants reach 9-10 months of age.

Most previous studies conducted in Japan have had methodological limitations, including in relation to the sample size, area of the study, and the study periods.^14^^,^^15^ The present survey was conducted with the participation of with several medical centers, with a large number of subjects. Also, the surveys were conducted twice in the same subjects. Moreover, the HADS was used to estimate the psychological symptoms in order to avoid, as much as possible, the confounding effects of the participants hiding their physical conditions.

In interpreting the present results, the following factors should be taken into consideration. In this study, only 53 % of the participants responded to both the surveys. The reasons for the refusal to participate in the first and/or second survey were not investigated. Considering the possibility of the non-responders including those with severe psychological distress, the prevalence of maternal psychological distress may have been underestimated in the present study. From the information collected at the time of recruitment of the subjects, the women who quit the study at any point were more likely to be young, have a weaker educational background and a lower annual income than those who responded to both surveys (Sato Y: unpublished observations). As to the prevalence of psychological distress, there was no significant difference in the prevalence of psychological distress in the first surveys between participants who responded only to the first survey and those who responded to both surveys.

Various situations and multiple factors influence the development of psychological symptoms in a mother.^16^^,^^17^ In Japan, the social environment related to child-rearing has changed during the five years.^15^ Thus, further surveys are proposed to examine factors related to maternal psychological distress in recent years in the Japanese population.

## References

[1] Murray L, Fiori-Cowley A, Hooper R, Cooper P. The impact of postnatal depression and associated adversity on early mother-infant interactions and later infant outcome. Child Dev 1996; 67: 2512-26. 10.2307/1131637 9022253

[2] Beck CT. The effects of postpartum depression on child development: a meta-analysis. Arch Psychiatr Nurs 1998; 12: 12-20. 10.1016/S0883-9417(98)80004-6 9489170

[3] Minkovitz CS, Strobino D, Scharfstein D, Hou W, Miller T, Mistry KB, . Maternal depressive symptoms and children’s receipt of health care in the first three years of life. Pediatrics 2005; 115: 306-14. 10.1542/peds.2004-0341 15687437

[4] Barnett B, Schaafsma M, Guzman A, Parker G. Maternal anxiety: a 5-year review of an intervention study. J Child Psychol Psychiatry 1991; 32: 423-38. 10.1111/j.1469-7610.1991.tb00321.x 2061363

[5] Mother’s & Children’s Health & Welfare Association. Maternal and Child Health Statistics of Japan. Mother’s & Children’s Health Organization, Tokyo, 2006: 151-9.

[6] Healthy Parents and Children 21 Committee Report (Sukoyaka Oyako 21 Kentokai Hokokusho). J Child Health 2001; 60: 5-33. (in Japanese)

[7] Sato Y, Kato T, Ito R, Gu YH, Kakee N Women’s satisfaction and effective care at childbirth. J Child Health 2007; 66: 465-71. (in Japanese)

[8] Zigmond AS, Snaith RP. The Hospital Anxiety and Depression Scale. Acta Psychiatr Scand 1983; 67: 361-70. 10.1111/j.1600-0447.1983.tb09716.x 6880820

[9] Kitamura T. Hospital Anxiety and Depression Scale (HAD Scale). Seishinkashindangaku 4, Nippon Hyoron Sha Co., Ltd, Tokyo, 1993: 371-2. (in Japanese)

[10] Higashi A, Yashiro H, Kiyota K, Inokuchi H, Hatta H, Fujita K, . Validation of the hospital anxiety and depression scale in a gastro-intestinal clinic. Nippon Shokakibyo Gakkai Zasshi 1996; 93: 884-92. (in Japanese) 8986079

[11] Hatta H, Higashi A, Yashiro H, Ozawa K, Hayashi K, Kiyota K, A validation of the Hospital Anxiety and Depression Scale. Jpn J Psychosom Med (Shinshin Igaku) 1998; 38: 309-15. (in Japanese)

[12] Abiodun OA. A validity study of the Hospital Anxiety and Depression Scale in general hospital units and a community sample in Nigeria. Br J Psychiatry 1994; 165: 669-72. 10.1192/bjp.165.5.669 7866683

[13] Spinhoven P, Ormel J, Sloekers PP, Kempen GI, Speckens AE, Van Hemert AM. A validation study of the Hospital Anxiety and Depression Scale (HADS) in different groups of Dutch subjects. Psychol Med 1997; 27: 363-70. 10.1017/S0033291796004382 9089829

[14] Arimoto A, Murashima S. Child-rearing anxiety and its correlates among Japanese mothers screened at 18-month Infant Health Checkups. Public Health Nurs 2007; 24: 101-10. 10.1111/j.1525-1446.2007.00614.x 17319882

[15] Shimada M, Sugimoto M, Agata T, Nitta N, Seki K, Ohashi K, Nationwide Survey on Maternal Anxiety in one-month postpartum, Needs for childrearing supports and environment at five years after Healthy Parents and Children 21 Program. J Child Health 2006; 65: 752-62. (in Japanese)

[16] Dankner R, Goldberg RP, Fisch RZ, Crum RM. Cultural elements of postpartum depression. A study of 327 Jewish Jerusalem women. J Reprod Med 2000; 45: 97-104. 10710738

[17] Howell EA, Mora PA, Horowitz CR, Leventhal H. Racial and ethnic differences in factors associated with early postpartum depressive symptoms. Obstet Gynecol 2005; 105: 1442-50. 10.1097/01.AOG.0000164050.34126.37 15932842 PMC4302723

